# Correction: Investigation of the relationship between sensorineural hearing loss and associated comorbidities in patients with chronic kidney disease: A nationwide, population-based cohort study

**DOI:** 10.1371/journal.pone.0247371

**Published:** 2021-02-17

**Authors:** The PLOS ONE Staff

The images for Figs 1 and 2 are incorrectly switched. The image that appears as Fig 1 should be Fig 2, and the image that appears as Fig 2 should be Fig 1. The figure captions appear in the correct order. The publisher apologizes for the error.

**Fig 1:**
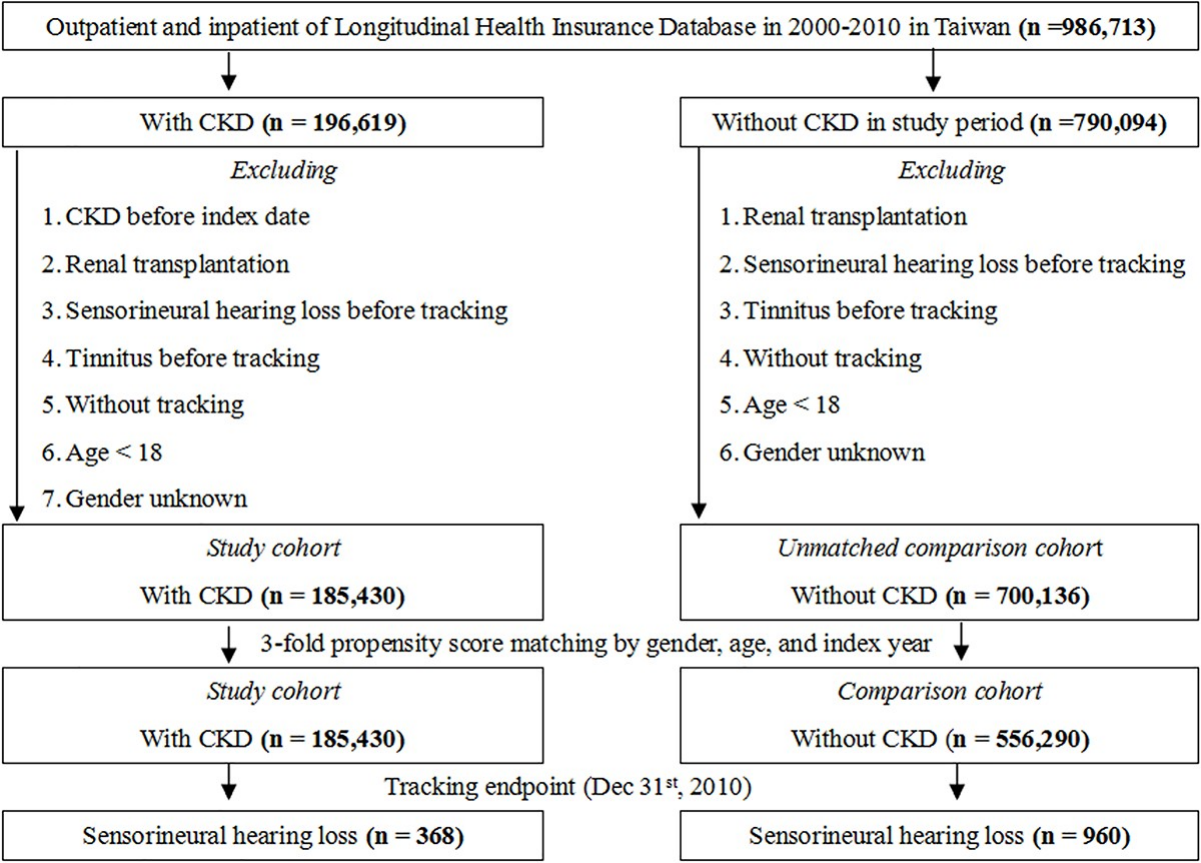
The flowchart of study sample selection.

**Fig 2:**
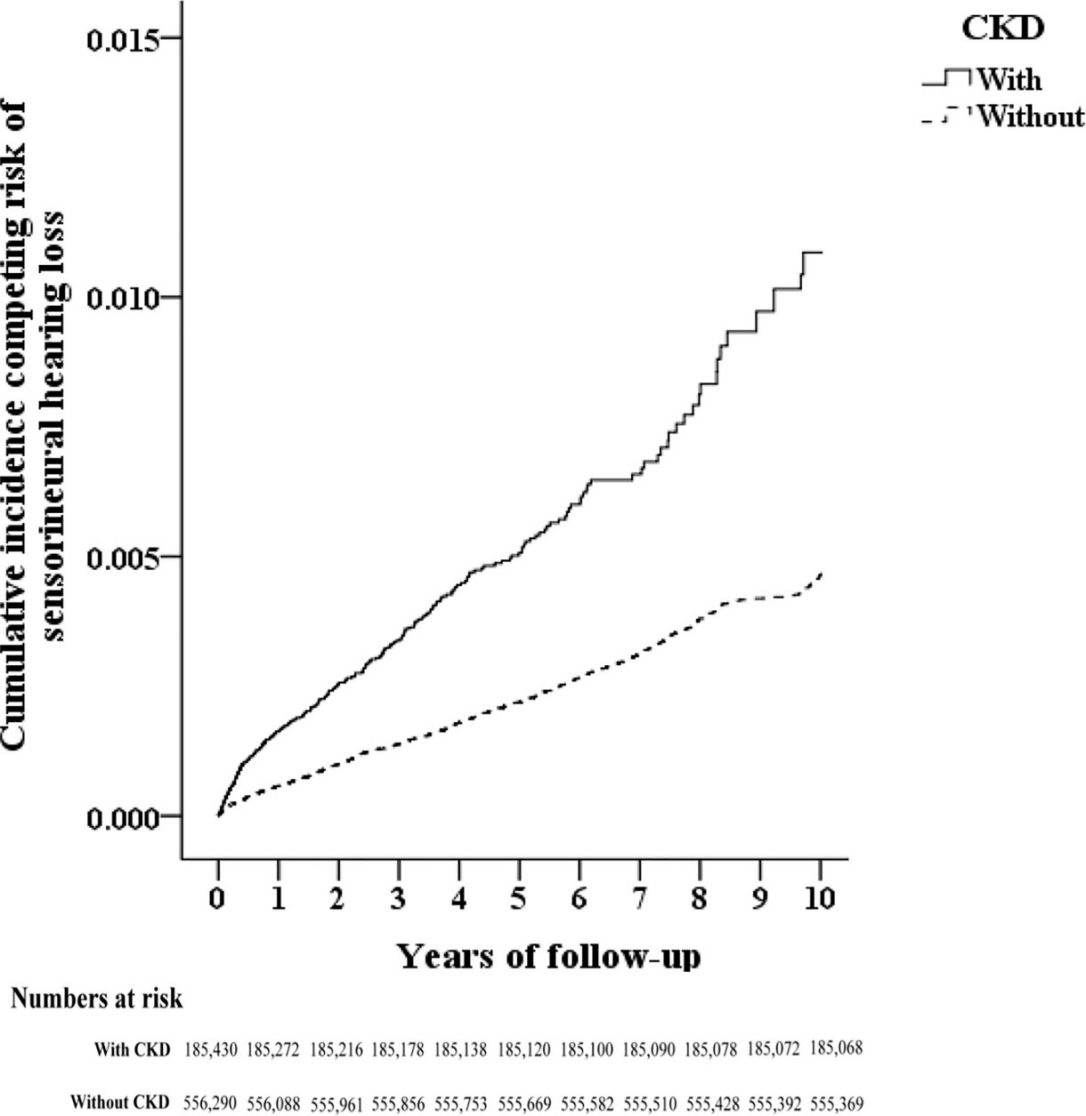
The cumulative incidence competing risk (CICR) method for the incidence of sensorineural hearing loss among patients aged 18 and over stratified by CKD (*p* < .001).
