# Supplementary material for: Investigation of the relationship between sensorineural hearing loss and associated comorbidities in patients with chronic kidney disease: A nationwide, population-based cohort study
Source: PLoS One. 2020 Sep 11;15(9):e0238913. doi: 10.1371/journal.pone.0238913 (PMC7485846; doi:10.1371/journal.pone.0238913)
Supplement: Attachment — Submitted filename: 1081120 response to reviewers (PLOS ONE).docx [file pone.0238913.s001.docx]

**November 20, 2019**

**PLOS ONE**

**Editor in Chief**

**Dear** **professor Jo George:**

Enclosed please find the manuscript, entitled “**Investigation of the relationship between sensorineural hearing loss and associated comorbidities in patients with chronic kidney disease: a nationwide, population-based cohort study”** by Wu KL et al for publication as a **c**linical research study in ***PLOS ONE.***

The manuscript represents an original clinical research study that is not under consideration for publication elsewhere until your decision has been made for its acceptability for ***PLOS ONE.*** The manuscript has been seen and approved by all authors. There are no potential conflicts of interest, including full disclosure of any financial arrangement. All authors had full access to all data. *Po-Jen Hsiao and Wu-Chien Chien* takes responsibility for the integrity of the data. The authors transfer all copyright ownership of the above manuscript to ***PLOS ONE*** in the event the work is published.

As you are aware, we submitted my research to you a while ago. We went back to the sources of information. In particular all of the code we wrote is available and I have included multiple links throughout the paper to the appropriate code repositories.

We believe that the manuscript is now suitable for publication in ***PLOS ONE.***

We look forward to your prompt reply.

Yours sincerely,

Po-Jen Hsiao*, Assistant Professor

No.168, Zhongxing Rd., Longtan Dist., Taoyuan City, 325, Taiwan, Republic of China (R.O.C.)

E-mail：a2005a660820@yahoo.com.tw

Wu-Chien Chien*, Associate Professor

No.325, Section 2, Cheng-Kung Road, Neihu District, Taipei City, 11490, Taiwan, Republic of China (R.O.C.)

Tel: 886-2-87923311 ; Fax: 886-2-87927235

E-mail：chienwu@ndmctsgh.edu.tw

**Response to Reviewer 1:**

**General comments**

**In this study, the authors determined the association between CKD and SNHL in the Longitudinal Health Insurance Database of the Taiwan National Health Insurance Research Database. They found CKD is independently associated with the risk of developing SNHL. While the findings are interesting, the some of the analysis are inappropriate.**

**Remedy:** We are grateful for your positive comments and careful reading of our manuscript. We have revised our manuscript according to your comments and the corresponding changes in the revised manuscript have been highlighted in red font and underlined. The itemized responses to your comments are below

**Specific Comments:**

**Point 1. Dr. Wu et al. performed a study to demonstrate people with diagnosis of “CKD” had higher risk of SNHL using the NHIRD which is already demonstrated in the following articles:**

**Indian J Otolaryngol Head Neck Surg. 2012 Dec;64(4):356-9.**

**BMC Nephrol. 2015 Sep 16;16:154. doi: 10.1186/s12882-015-0151-0.**

**Ren Fail. 2018 Nov;40(1):170-174**

**However, lack of advanced information and only the epidemiology data and hazard ratio were provided in this study. The result may not tell the casual effect of CKD itself or the aminoglycoside/ loop diuretics contributed to SNHL.**

**Remedy:** We thank the valuable comment about the association between SNHL (sensorineural hearing loss) and CKD. The CKD patients often have multiple systemic dysfunctions such as cardiovascular, lung, liver, metabolic, brain, immune system, and chronic inflammation further resulting in various comorbidities. So we designed advanced study to identify the risk of sensorineural hearing loss in CKD patients with associated comorbidities. The increased risk of developing SNHL in patients with CKD and comorbidities such as heart failure, liver cirrhosis, type 2 DM, hypertension, and COPD (table 4) was demonstrated. The synergetic effect between these medical conditions may aggravate the developing sensorineural hearing loss in our epidemiology data. The inter-organ crosstalk may be a key point of sensorineural hearing loss by chronic inflammation, oxidative stress, and sympathetic nerve hyperactivity reported in previous studies.

In addition, the ototoxicity of aminoglycoside and loop diuretics for sensorineural hearing loss in CKD patients has been investigated without significance. The nephrotoxic and ototoxic agents have been used by physicians with caution in CKD patients. The associated comorbidities in CKD patients should be considered for the increased risk of sensorineural hearing loss.

**Point 2. The author did not define the variable "season" clearly in the method section. Whether it means the "season" when SNHL is made is not clear. In addition, the relationship between the "season" and SNHL should be discussed, since the relationship between season and certain kinds of disease (e.g. flu, pneumonia, kidney stones) are quite reasonable, but the linkage of SNHL arcoss the whole year is not very straightforward. How do you explain the difference in season and the risk of SNHL?**

**Remedy:** We completely agree with this comment. The influence of "season" for SNHL cannot be well estimated in our study, so we deleted the description.

**Point 3. The patients' CKD severity may be different when they were diagnosed with the ICD code 585. This retrospective cohort may have problems of lead-time bias, therefore the time at risk may not be corrected because the starting point of CKD is different in each patient. It could be assumed that some patients entered this database with advenced CKD initially, and therefore their time-to-SNHL is significantly underestimated., and the risk of SNHL may be over-estimated. The author need to clarify this possible bias.**

**Remedy:** We completely agree with this invaluable comment. The limitation was descripted “Secondly, although NHIRD coding in recording diseases has been validated [12,13], there is no data regarding laboratory parameters and severity of hearing loss for coding the accuracy of diseases. Nevertheless, in an attempt to increase the validity of diagnosis, this study matched diagnosis to three indices and restricted the diagnosis of hearing loss by an otorhinolaryngologist only. However, the influence of the severity of CKD on the severity of hearing loss cannot be analyzed.” (discussion: line 259-265)

**Point4. Materials and Methods**

**4-1. Data Sources**

**Please explicitly point out the database of “LHID 2000”.**

**Remedy:** We greatly appreciate this comment. We descript “The Bureau of National Health Insurance randomly reviews the records of 1 in 100 ambulatory care visits and 1 in 20 in-patient claims to verify the accuracy of the diagnoses. The accuracy and validity of the diagnoses have been demonstrated [12,13]. Therefore, the NHIRD was used as the data source. The study used data from 2000 to 2010 from the Longitudinal NHIRD to analyze the risk of developing SNHL in patients with CKD.” (Please see Material and Methods, section: line: 109-114)

**4-2. They should indicate the index date of CKD group. For example, the first diagnosis of CKD during 2000-2010 was assigned as the index date of CKD group.**

**Remedy:** We agree with this comment. The changed description was “A 10-year retrospective matched-cohort study was designed and 986,713 consecutive patients from January 1, 2000 to December 31, 2010 were enrolled. Patients newly diagnosed with CKD (ICD-9-CM codes, 585–586) before 1999, who had received a renal transplantation (ICD-9-CM code, V42.0), who had SNHL (ICD-9-CM code, 389.1) or tinnitus (ICD-9-CM code 388.3) before tracking, who aged <18 years, and who were without tracking or of unknown gender were excluded. After matching the CKD participants with thrice comparison subjects (index year, month, gender and age), 185,430 patients with first diagnosis of CKD and 556,290 participants without CKD were included in the CKD and comparison groups, respectively.” (Please see Material and Methods, section: Study design and participants, line 120-129)

**4-3. They should make it more clear as to how they design and generate the control group.**

**Remedy:** We completely agree with this important comment. We have changed the description in the materials and methods” After matching the CKD participants with thrice comparison subjects (index year, month, gender and age), 185,430 patients with CKD and 556,290 participants without CKD were included in the CKD and comparison groups, respectively.” (Material and Methods, section: Study design and participants, line 126-129)

**4-4. It is suggested to replace “control” with “comparison” because the term “control” is commonly used in retrospective case-control study.**

**Remedy:** Thank you for the reminder. We used comparison group for participants without CKD in our text.

**4-5. The ascertainment of diagnosis of SNHL was nicely reported.**

**Remedy:** Thank you for the reminder. We have changed the description “The occurrence of SNHL (ICD-9-CM code, 389.1) that was diagnosed by otorhinolaryngologists at least thrice, continued for at least 4 weeks, and tracked until December 31, 2010.” (Please see Material and Methods, section: Study design and participants, line 129-131)

**Point 5. Statistical analysis**

**5-1. Do not use t-test or chi-square test in this large-scale study, please use standardized difference / standardized mean difference instead.**

**Remedy:** The authors thank to the valuable suggestion and amend our manuscript as the following as well. **“**…. We present **standardized difference and standardized mean difference for** categorical and continuous variable distributions, respectively. Meanwhile ….“

| Table 1. Characteristics of study in the baseline | | | | | | | | | | | |
| --- | --- | --- | --- | --- | --- | --- | --- | --- | --- | --- | --- |
|  | Overall  (n = 741,720) | |  | With CKD  (n= 185,430) | |  | Without CKD  (n= 556,290) | | *P* | Standardized difference | Standardized mean difference |
| Variables | n | % |  | n | % |  | n | % |  |  |  |
| Gender |  |  |  |  |  |  |  |  | 0.999 | 0.000 | 0.000 |
| Male | 402,880 | 54.32 |  | 100,720 | 54.32 |  | 302,160 | 54.32 |  |  |  |
| Female | 338,840 | 45.68 |  | 84,710 | 45.68 |  | 254,130 | 45.68 |  |  |  |
| Age (years) | 67.31 ± 13.55 | |  | 67.30 ± 14.20 | |  | 67.31 ± 13.33 | | 0.783 | -1.995 | -0.363 |
| Age groups (yrs) |  |  |  |  |  |  |  |  | 0.999 | 0.000 | 0.000 |
| 18-29 | 10,226 | 1.38 |  | 2,559 | 1.38 |  | 7,667 | 1.38 |  |  |  |
| 30-39 | 24,428 | 3.29 |  | 6,107 | 3.29 |  | 18,321 | 3.29 |  |  |  |
| 40-49 | 63,152 | 8.51 |  | 15,788 | 8.51 |  | 47,364 | 8.51 |  |  |  |
| 50-59 | 111,160 | 14.99 |  | 27,790 | 14.99 |  | 83,370 | 14.99 |  |  |  |
| ≧60 | 532,744 | 71.83 |  | 133,186 | 71.83 |  | 399,558 | 71.83 |  |  |  |
| Insured premium (NT$) |  |  |  |  |  |  |  |  | <0.001^***^ | -0.032 | -0.002 |
| <18,000 | 732,832 | 98.80 |  | 183,435 | 98.92 |  | 549,397 | 98.75 |  |  |  |
| 18,000-34,999 | 7,780 | 1.05 |  | 1,788 | 0.96 |  | 5,992 | 1.08 |  |  |  |
| ≧35,000 | 1,158 | 0.16 |  | 207 | 0.11 |  | 951 | 0.17 |  |  |  |
| Comorbidities |  |  |  |  |  |  |  |  |  |  |  |
| HTN | 156,363 | 21.08 |  | 42,234 | 22.78 |  | 114,129 | 20.52 | <0.001^***^ | 0.023 | 0.001 |
| T2DM | 144,996 | 19.55 |  | 63,178 | 34.07 |  | 81,818 | 14.71 | <0.001^***^ | 0.202 | 0.001 |
| CHF | 38,776 | 5.23 |  | 21,441 | 11.56 |  | 17,335 | 3.12 | <0.001^***^ | 0.084 | 0.001 |
| Stroke | 69,462 | 9.36 |  | 13,180 | 7.11 |  | 56,282 | 10.12 | <0.001^***^ | -0.030 | -0.001 |
| COPD | 66,119 | 8.91 |  | 11,927 | 6.43 |  | 54,192 | 9.74 | <0.001^***^ | -0.003 | -0.001 |
| Liver cirrhosis | 41,886 | 5.65 |  | 10,910 | 5.88 |  | 30,976 | 5.57 | <0.001^***^ | 0.003 | 0.001 |
| Meniere's disease | 13,890 | 1.87 |  | 1,434 | 0.77 |  | 12,456 | 2.24 | <0.001^***^ | -0.015 | <0.001 |
| CCI_R | 1.24 ± 2.06 | |  | 2.63 ± 1.61 | |  | 0.77 ± 1.98 | | <0.001^***^ | 1.942 | 0.005 |
| Medications |  |  |  |  |  |  |  |  |  |  |  |
| Aminoglycoside | 4,585 | 0.62 |  | 600 | 0.32 |  | 3,985 | 0.72 | <0.001^***^ | -0.003 | -0.001 |
| Loop diuretics | 4,733 | 0.64 |  | 1,634 | 0.88 |  | 3,099 | 0.56 | <0.001^***^ | 0.004 | 0.001 |

**5-2. In table 2-4, the authors calculated the adjust HR, and stated that they adjust for variables in the table. Is that means you calculate the adjusted HR for those with/withour CKD b adjusting ALL variables in the table ? (adjusted for age, sex, HTN, DM, CHF, stroke, COPD, liver cirrhosis, Season, urbanization level, level of care and insurance premium). The interaction between certain comorbidities may exist and did you test the co-linearity between the comorbidites?**

**Remedy:**” Thank you for this suggestion. The authors re-analyze and amend totally tables in the manuscript as the following as well. The increased values of the adjusted HRs indicated synergetic effect of CKD and comorbidities for SNHL.

| Table 2. Factors of sensorineural hearing loss by using Cox regression and Fine & Gray's competing risk model | | | | | | | | | |
| --- | --- | --- | --- | --- | --- | --- | --- | --- | --- |
|  | No competing risk in the model | | | |  | Competing risk in the model | | | |
|  | Adjusted HR | 95% CI | 95% CI | *P* |  | Adjusted HR | 95% CI | 95% CI | *P* |
| CKD *(Reference: Without)* | 3.19 | 2.80 | 3.64 | <0.001^***^ |  | 3.42 | 3.01 | 3.90 | <0.001^***^ |
| Gender *(Reference: Female)* | 1.30 | 1.16 | 1.45 | <0.001^***^ |  | 1.32 | 1.19 | 1.48 | <0.001^***^ |
| Age groups (yrs) *(Reference: 18-29)* |  |  |  |  |  |  |  |  |  |
| 30-39 | 0.52 | 0.19 | 1.40 | 0.192 |  | 0.53 | 0.20 | 1.43 | 0.205 |
| 40-49 | 0.95 | 0.45 | 2.21 | 0.896 |  | 0.99 | 0.42 | 2.30 | 0.969 |
| 50-59 | 0.99 | 0.44 | 2.24 | 0.968 |  | 1.05 | 0.46 | 2.37 | 0.931 |
| ≧60 | 0.12 | 0.51 | 2.53 | 0.790 |  | 1.28 | 0.57 | 2.86 | 0.568 |
| Comorbidities *(Reference: Without)* |  |  |  |  |  |  |  |  |  |
| HTN | 0.89 | 0.79 | 1.01 | 0.052 |  | 0.84 | 0.75 | 0.95 | 0.009^**^ |
| T2DM | 0.86 | 0.76 | 1.01 | 0.051 |  | 0.85 | 0.75 | 1.00 | 0.046^*^ |
| CHF | 0.52 | 0.40 | 0.69 | <0.001^***^ |  | 0.55 | 0.42 | 0.73 | <0.001^***^ |
| Stroke | 1.49 | 1.29 | 1.74 | <0.001^***^ |  | 1.52 | 1.31 | 1.77 | <0.001^***^ |
| COPD | 1.22 | 1.04 | 1.45 | 0.026 ^*^ |  | 1.21 | 1.04 | 1.53 | 0.019 ^*^ |
| Liver cirrhosis | 1.13 | 0.89 | 1.45 | 0.338 |  | 1.19 | 0.94 | 1.55 | 0.192 |
| Meniere's disease | 8.51 | 7.11 | 10.17 | <0.001^***^ |  | 7.81 | 6.53 | 9.34 | <0.001^***^ |
| CCI_R *(Reference: Without)* | 0.95 | 0.93 | 0.98 | 0.006^**^ |  | 0.97 | 0.94 | 1.00 | 0.027^*^ |
| Medications |  |  |  |  |  |  |  |  |  |
| Aminoglycoside | 0.37 | 0.12 | 1.14 | 0.267 |  | 0.37 | 0.12 | 1.18 | 0.143 |
| Loop diuretics | 0.19 | 0.05 | 0.75 | <0.001^***^ |  | 0.21 | 0.05 | 0.86 | 0.005^*^ |
| Adjusted HR (hazard ratio): Adjusted variables listed in the table; CI = confidence interval; ^*^*P* < 0.05, ^**^*P* < 0.01, ^***^*P* < 0.001. CKD: chronic kidney disease, HTN: hypertension, T2DM: type 2 diabetes mellitus, CHF: heart failure, COPD: chronic obstructive pulmonary disease. | | | | | | | | | |

| Table 3. Factors of sensorineural hearing loss stratified by with / without hemodialysis by using Cox regression and Fine & Gray's competing risk model | | | | | | | | | | | | | | |
| --- | --- | --- | --- | --- | --- | --- | --- | --- | --- | --- | --- | --- | --- | --- |
|  |  |  |  |  |  | No competing risk in the model | | | |  | Competing risk in the model | | | |
|  | Populations | Events | PYs | Rate |  | Adjusted HR | 95% CI | 95% CI | *P* |  | Adjusted HR | 95% CI | 95% CI | *P* |
| Without CKD | 556,290 | 960 | 1,168,647.66 | 82.146 |  | *Reference* |  |  |  |  | *Reference* |  |  |  |
| With CKD | 185,430 | 368 | 334,193.43 | 110.116 |  | 3.19 | 2.80 | 3.64 | <0.001^***^ |  | 3.42 | 3.01 | 3.90 | <0.001^***^ |
| without hemodialysis | 98,069 | 198 | 200,431.46 | 98.787 |  | 1.31 | 0.99 | 3.01 | 0.058 |  | 1.40 | 1.01 | 3.23 | 0.046^*^ |
| with hemodialysis | 87,361 | 170 | 133,761.97 | 127.091 |  | 5.52 | 2.82 | 10.98 | <0.001^***^ |  | 5.92 | 3.03 | 11.79 | <0.001^***^ |
| PYs = Person-years; Rate: per 100,000 PYs; Adjusted HR (hazard ratio): Adjusted variables listed in Table 1; CI = confidence interval; ^*^*P* < 0.05, ^**^*P* < 0.01, ^***^*P* < 0.001. CKD: chronic kidney disease. | | | | | | | | | | | | | | |

| Table 4. Factors of sensorineural hearing loss stratified by variables listed in the table by using Fine & Gray's competing risk model | | | | | | | | | | | | | |
| --- | --- | --- | --- | --- | --- | --- | --- | --- | --- | --- | --- | --- | --- |
|  |  | With CKD | | |  | With CKD | | |  | With CKD *vs.* Without CKD *(Reference)* | | | |
| Variables | Strarified | Events | PYs | Rate |  | Events | PYs | Rate |  | Adjusted HR | 95% CI | 95% CI | *P* |
|  | Overall | 368 | 334,193.43 | 110.12 |  | 960 | 1,168,647.66 | 82.15 |  | 3.42 | 3.01 | 3.90 | <0.001^***^ |
| Gender | Male | 198 | 174,093.39 | 113.73 |  | 595 | 629,014.38 | 94.59 |  | 2.98 | 2.50 | 3.54 | <0.001^***^ |
|  | Female | 170 | 160,100.04 | 106.18 |  | 365 | 539,633.28 | 67.64 |  | 4.31 | 3.44 | 5.15 | <0.001^***^ |
| Age groups (yrs) | 18-29 | 6 | 2,450.49 | 244.85 |  | 0 | 4,350.17 | 0.00 |  | ∞ | - | - | 0.897 |
|  | 30-39 | 6 | 8,722.15 | 68.79 |  | 5 | 15,192.58 | 32.91 |  | 3.37 | 0.77 | 14.82 | 0.724 |
|  | 40-49 | 32 | 24,547.77 | 130.36 |  | 23 | 69,293.14 | 33.19 |  | 4.30 | 2.34 | 7.89 | <0.001^***^ |
|  | 50-59 | 40 | 54,197.86 | 73.80 |  | 99 | 305,306.51 | 32.43 |  | 1.61 | 1.08 | 2.41 | 0.041^*^ |
|  | ≧60 | 284 | 244,275.16 | 116.26 |  | 833 | 744,505.26 | 111.89 |  | 3.67 | 3.18 | 4.23 | <0.001^***^ |
| HTN | Without | 216 | 196,821.62 | 109.74 |  | 677 | 823,506.35 | 82.21 |  | 3.36 | 2.85 | 3.99 | <0.001^***^ |
|  | With | 152 | 137,371.81 | 110.65 |  | 283 | 345,141.31 | 82.00 |  | 3.67 | 2.95 | 4.62 | <0.001^***^ |
| T2DM | Without | 244 | 226,763.67 | 107.60 |  | 782 | 902,618.82 | 86.64 |  | 3.24 | 2.77 | 3.77 | <0.001^***^ |
|  | With | 124 | 107,429.76 | 115.42 |  | 178 | 266,028.84 | 66.91 |  | 3.98 | 3.08 | 4.85 | <0.001^***^ |
| CHF | Without | 338 | 302,066.10 | 111.90 |  | 936 | 1,085,462.92 | 86.23 |  | 3.29 | 2.87 | 3.76 | <0.001^***^ |
|  | With | 30 | 32,127.33 | 93.38 |  | 24 | 83,184.74 | 28.85 |  | 7.48 | 4.08 | 13.78 | <0.001^***^ |
| Stroke | Without | 329 | 303,790.75 | 108.30 |  | 793 | 1,031,091.16 | 76.91 |  | 3.48 | 3.03 | 4.02 | <0.001^***^ |
|  | With | 39 | 30,402.68 | 128.28 |  | 167 | 137,556.50 | 121.40 |  | 2.99 | 2.06 | 4.35 | <0.001^***^ |
| COPD | Without | 342 | 313,272.56 | 109.17 |  | 830 | 1,024,602.66 | 81.01 |  | 3.39 | 2.96 | 3.90 | <0.001^***^ |
|  | With | 26 | 20,920.87 | 124.28 |  | 130 | 144,045.00 | 90.25 |  | 3.45 | 2.23 | 5.32 | <0.001^***^ |
| Liver cirrhosis | Without | 344 | 317,482.25 | 108.35 |  | 915 | 1,108,300.52 | 82.56 |  | 3.39 | 2.97 | 3.88 | <0.001^***^ |
|  | With | 24 | 16,711.18 | 143.62 |  | 45 | 60,347.14 | 74.57 |  | 4.12 | 2.42 | 7.03 | <0.001^***^ |
| Meniere's disease | Without | 356 | 331,468.23 | 107.40 |  | 834 | 1,131,989.89 | 73.68 |  | 3.58 | 3.13 | 4.10 | <0.001^***^ |
|  | With | 12 | 2,725.20 | 440.33 |  | 126 | 36,657.77 | 343.72 |  | 1.64 | 1.01 | 3.12 | 0.047^*^ |
| Aminoglycoside | Without | 366 | 332,526.32 | 110.07 |  | 953 | 1,161,049.27 | 82.08 |  | 3.41 | 3.00 | 3.92 | <0.001^***^ |
|  | With | 2 | 1,667.11 | 119.97 |  | 7 | 7,598.39 | 92.12 |  | 3.33 | 2.83 | 3.83 | <0.001^***^ |
| Loop diuretics | Without | 367 | 331,401.74 | 110.74 |  | 957 | 1,157,557.93 | 82.67 |  | 3.42 | 3.00 | 3.93 | <0.001^***^ |
|  | With | 1 | 2,791.69 | 35.82 |  | 3 | 11,089.73 | 27.05 |  | 3.39 | 2.96 | 3.86 | <0.001^***^ |
| PYs = Person-years; Rate: per 100,000 PYs; Adjusted HR (hazard ratio): Adjusted variables listed in the table; CI = confidence interval; ^*^*P* < 0.05, ^**^*P* < 0.01, ^***^*P* < 0.001. CKD: chronic kidney disease, HTN: hypertension, T2DM: type 2 diabetes mellitus, CHF: heart failure, COPD: chronic obstructive pulmonary disease. | | | | | | | | | | | | | |

**5-3. Please replace ‘multivariate’ with ‘multivariable’ throughout the text.**

**Remedy:** Thank you for this command. We have revised the description “Multivariable cox proportional hazards regression analysis was used to determine the risk of developing hearing loss.” (line 149-150).

**5-4. “The extent of CKD was estimated using the Charlson Comorbidity Index after removing the aforementioned comorbidities (CCI_R)”. This sentence is not clear. Please explain what components were included in CCI and what comorbidities they excluded from the CCI and why doing this can measure the extent of CKD.**

**Remedy:** Thank you for this suggestion. The Charlson Comorbidities Index is as follows: 1 each: Myocardial infarct, congestive heart failure, peripheral vascular disease, dementia, cerebrovascular disease, chronic lung disease, connective tissue disease, ulcer, chronic liver disease, diabetes, 2 each: Hemiplegia, moderate or severe kidney disease, diabetes with end organ damage, tumor, leukemia, lymphoma, 3 each: Moderate or severe liver disease, and 6 each: Malignant tumor, metastasis, AIDS. We have revised the description in method “ The 17 weighted indicators of comorbidities were used to calculate the Charlson Comorbidity Index (CCI) [15]. The extent of CKD was estimated using the CCI after removing the aforementioned comorbidities (CCI_R).” (please see Material and Methods, line 156-159)

Reference:

15. Charlson ME, Pompei P, Ales KL, MacKenzie CR. A new method of classifying prognostic comorbidity in longitudinal studies: development and validation. J Chronic Dis. 1987;40:373-83.

**5-5. Had better provide a sensitivity analysis that use death during the follow up as a competing risk (Fine and Gray subdistribution hazard model). The cumulative incidence was downgraded more when the competing risk was more common. As known, CKD patients are prone to have higher mortality rates, it is important to confirm whether CKD is a risk factor of SNHL when considering the competing risk of mortality.**

**Remedy:** Thank you for this invaluable comment. The description of competing risk model has been added in method, “We also performed a competing-risks regression (Fine-Gray model) because CKD risk might compete with the risk of death [14].” (please see Material and Methods, section: Statistical analysis line: 152-154)

Reference:

14. Blanche P, Proust-Lima C, Loubère L, Berr C, Dartigues JF, Jacqmin-Gadda H. Quantifying and comparing dynamic predictive accuracy of joint models for longitudinal marker and time-to-event in presence of censoring and competing risks. Biometrics. 2015;71(1):102-13.

**Point 6. Results**

**6-1. Line 153-159**

**Remove all the p values in the text, provide standardized difference / standardized mean difference instead.**

**Remedy:** The authors thank to the valuable suggestion and amend our manuscript as the following as well. The changed description was theat “…. We present **standardized difference and standardized mean difference for** categorical and continuous variable distributions, respectively. Meanwhile ….”.

**6-2. Table 1**

**The table is very long. Please leave only one row when the number of category was 2 (i.e., gender, stroke).**

**Level of care? Provide definition of this variable. How do they define that control subjects were followed at which level of institution?**

**The distribution of insurance premium is very extreme. Why the numbers were so rare in the 18,000–34,999 and ≧35,000 groups?**

**Remedy:** Thank you for this comment. We had revised the table 1. For covering most population, the insurance premium was set in affordable range by government policy. The insurance premium was paid to the Bureau of National Health Insurance by people according the level of income difference.

| Table 1. Characteristics of study in the baseline | | | | | | | | | | | |
| --- | --- | --- | --- | --- | --- | --- | --- | --- | --- | --- | --- |
|  | Overall  (n = 741,720) | |  | With CKD  (n= 185,430) | |  | Without CKD  (n= 556,290) | | *P* | Standardized difference | Standardized mean difference |
| Variables | n | % |  | n | % |  | n | % |  |  |  |
| Gender |  |  |  |  |  |  |  |  | 0.999 | 0.000 | 0.000 |
| Male | 402,880 | 54.32 |  | 100,720 | 54.32 |  | 302,160 | 54.32 |  |  |  |
| Female | 338,840 | 45.68 |  | 84,710 | 45.68 |  | 254,130 | 45.68 |  |  |  |
| Age (years) | 67.31 ± 13.55 | |  | 67.30 ± 14.20 | |  | 67.31 ± 13.33 | | 0.783 | -1.995 | -0.363 |
| Age groups (yrs) |  |  |  |  |  |  |  |  | 0.999 | 0.000 | 0.000 |
| 18-29 | 10,226 | 1.38 |  | 2,559 | 1.38 |  | 7,667 | 1.38 |  |  |  |
| 30-39 | 24,428 | 3.29 |  | 6,107 | 3.29 |  | 18,321 | 3.29 |  |  |  |
| 40-49 | 63,152 | 8.51 |  | 15,788 | 8.51 |  | 47,364 | 8.51 |  |  |  |
| 50-59 | 111,160 | 14.99 |  | 27,790 | 14.99 |  | 83,370 | 14.99 |  |  |  |
| ≧60 | 532,744 | 71.83 |  | 133,186 | 71.83 |  | 399,558 | 71.83 |  |  |  |
| Insured premium (NT$) |  |  |  |  |  |  |  |  | <0.001^***^ | -0.032 | -0.002 |
| <18,000 | 732,832 | 98.80 |  | 183,435 | 98.92 |  | 549,397 | 98.75 |  |  |  |
| 18,000-34,999 | 7,780 | 1.05 |  | 1,788 | 0.96 |  | 5,992 | 1.08 |  |  |  |
| ≧35,000 | 1,158 | 0.16 |  | 207 | 0.11 |  | 951 | 0.17 |  |  |  |
| Comorbidities |  |  |  |  |  |  |  |  |  |  |  |
| HTN | 156,363 | 21.08 |  | 42,234 | 22.78 |  | 114,129 | 20.52 | <0.001^***^ | 0.023 | 0.001 |
| T2DM | 144,996 | 19.55 |  | 63,178 | 34.07 |  | 81,818 | 14.71 | <0.001^***^ | 0.202 | 0.001 |
| CHF | 38,776 | 5.23 |  | 21,441 | 11.56 |  | 17,335 | 3.12 | <0.001^***^ | 0.084 | 0.001 |
| Stroke | 69,462 | 9.36 |  | 13,180 | 7.11 |  | 56,282 | 10.12 | <0.001^***^ | -0.030 | -0.001 |
| COPD | 66,119 | 8.91 |  | 11,927 | 6.43 |  | 54,192 | 9.74 | <0.001^***^ | -0.003 | -0.001 |
| Liver cirrhosis | 41,886 | 5.65 |  | 10,910 | 5.88 |  | 30,976 | 5.57 | <0.001^***^ | 0.003 | 0.001 |
| Meniere's disease | 13,890 | 1.87 |  | 1,434 | 0.77 |  | 12,456 | 2.24 | <0.001^***^ | -0.015 | <0.001 |
| CCI_R | 1.24 ± 2.06 | |  | 2.63 ± 1.61 | |  | 0.77 ± 1.98 | | <0.001^***^ | 1.942 | 0.005 |
| Medications |  |  |  |  |  |  |  |  |  |  |  |
| Aminoglycoside | 4,585 | 0.62 |  | 600 | 0.32 |  | 3,985 | 0.72 | <0.001^***^ | -0.003 | -0.001 |
| Loop diuretics | 4,733 | 0.64 |  | 1,634 | 0.88 |  | 3,099 | 0.56 | <0.001^***^ | 0.004 | 0.001 |

**6-3. Table 2**

**The table is very long. Please leave only one row when the number of category was 2 (i.e., gender, stroke).**

**Remedy:** Thank you for the reminder. We had revised the table 2.

| Table 2. Factors of sensorineural hearing loss by using Cox regression and Fine & Gray's competing risk model | | | | | | | | | |
| --- | --- | --- | --- | --- | --- | --- | --- | --- | --- |
|  | No competing risk in the model | | | |  | Competing risk in the model | | | |
|  | Adjusted HR | 95% CI | 95% CI | *P* |  | Adjusted HR | 95% CI | 95% CI | *P* |
| CKD *(Reference: Without)* | 3.19 | 2.80 | 3.64 | <0.001^***^ |  | 3.42 | 3.01 | 3.90 | <0.001^***^ |
| Gender *(Reference: Female)* | 1.30 | 1.16 | 1.45 | <0.001^***^ |  | 1.32 | 1.19 | 1.48 | <0.001^***^ |
| Age groups (yrs) *(Reference: 18-29)* |  |  |  |  |  |  |  |  |  |
| 30-39 | 0.52 | 0.19 | 1.40 | 0.192 |  | 0.53 | 0.20 | 1.43 | 0.205 |
| 40-49 | 0.95 | 0.45 | 2.21 | 0.896 |  | 0.99 | 0.42 | 2.30 | 0.969 |
| 50-59 | 0.99 | 0.44 | 2.24 | 0.968 |  | 1.05 | 0.46 | 2.37 | 0.931 |
| ≧60 | 0.12 | 0.51 | 2.53 | 0.790 |  | 1.28 | 0.57 | 2.86 | 0.568 |
| Comorbidities *(Reference: Without)* |  |  |  |  |  |  |  |  |  |
| HTN | 0.89 | 0.79 | 1.01 | 0.052 |  | 0.84 | 0.75 | 0.95 | 0.009^**^ |
| T2DM | 0.86 | 0.76 | 1.01 | 0.051 |  | 0.85 | 0.75 | 1.00 | 0.046^*^ |
| CHF | 0.52 | 0.40 | 0.69 | <0.001^***^ |  | 0.55 | 0.42 | 0.73 | <0.001^***^ |
| Stroke | 1.49 | 1.29 | 1.74 | <0.001^***^ |  | 1.52 | 1.31 | 1.77 | <0.001^***^ |
| COPD | 1.22 | 1.04 | 1.45 | 0.026 ^*^ |  | 1.21 | 1.04 | 1.53 | 0.019 ^*^ |
| Liver cirrhosis | 1.13 | 0.89 | 1.45 | 0.338 |  | 1.19 | 0.94 | 1.55 | 0.192 |
| Meniere's disease | 8.51 | 7.11 | 10.17 | <0.001^***^ |  | 7.81 | 6.53 | 9.34 | <0.001^***^ |
| CCI_R *(Reference: Without)* | 0.95 | 0.93 | 0.98 | 0.006^**^ |  | 0.97 | 0.94 | 1.00 | 0.027^*^ |
| Medications |  |  |  |  |  |  |  |  |  |
| Aminoglycoside | 0.37 | 0.12 | 1.14 | 0.267 |  | 0.37 | 0.12 | 1.18 | 0.143 |
| Loop diuretics | 0.19 | 0.05 | 0.75 | <0.001^***^ |  | 0.21 | 0.05 | 0.86 | 0.005^*^ |
| Adjusted HR (hazard ratio): Adjusted variables listed in the table; CI = confidence interval; ^*^*P* < 0.05, ^**^*P* < 0.01, ^***^*P* < 0.001. CKD: chronic kidney disease, HTN: hypertension, T2DM: type 2 diabetes mellitus, CHF: heart failure, COPD: chronic obstructive pulmonary disease. | | | | | | | | | |

**6-4. Table 3**

**Line 169-174**

**This analysis is very confusing. Does the hemodialysis (47.11%) occur after the diagnosis of CKD? If so, is it possible that hemodialysis occurs after SNHL?**

**Remady:** Thank your reminder. We longitudinally followed the patients with first-diagnosed CKD from 2000-2010 until the end point of sensorineural hearing loss. So the sensorineural hearing loss developed after the beginning of hemodialysis in the CKD patients with hemodialysis. (Please see Table 3 and figure 2)

| Table 3. Factors of sensorineural hearing loss stratified by with / without hemodialysis by using Cox regression and Fine & Gray's competing risk model | | | | | | | | | | | | | | |
| --- | --- | --- | --- | --- | --- | --- | --- | --- | --- | --- | --- | --- | --- | --- |
|  |  |  |  |  |  | No competing risk in the model | | | |  | Competing risk in the model | | | |
|  | Populations | Events | PYs | Rate |  | Adjusted HR | 95% CI | 95% CI | *P* |  | Adjusted HR | 95% CI | 95% CI | *P* |
| Without CKD | 556,290 | 960 | 1,168,647.66 | 82.146 |  | *Reference* |  |  |  |  | *Reference* |  |  |  |
| With CKD | 185,430 | 368 | 334,193.43 | 110.116 |  | 3.19 | 2.80 | 3.64 | <0.001^***^ |  | 3.42 | 3.01 | 3.90 | <0.001^***^ |
| without hemodialysis | 98,069 | 198 | 200,431.46 | 98.787 |  | 1.31 | 0.99 | 3.01 | 0.058 |  | 1.40 | 1.01 | 3.23 | 0.046^*^ |
| with hemodialysis | 87,361 | 170 | 133,761.97 | 127.091 |  | 5.52 | 2.82 | 10.98 | <0.001^***^ |  | 5.92 | 3.03 | 11.79 | <0.001^***^ |
| PYs = Person-years; Rate: per 100,000 PYs; Adjusted HR (hazard ratio): Adjusted variables listed in Table 1; CI = confidence interval; ^*^*P* < 0.05, ^**^*P* < 0.01, ^***^*P* < 0.001. CKD: chronic kidney disease. | | | | | | | | | | | | | | |

**Figure 2.** Kaplan-Meier for cumulative risk of sensorineural hearing loss among patients aged 18 and over stratified by CKD with log-rank test in competing risk model. CKD: chronic kidney disease.

**6-5. Table**

**Line 185-189**

**It is not meaningful to just report the HRs in each subgroup. They should provide interaction test (add one column in Table 4) and therefore report the HRs in each subgroup when the interaction was statistically significant.**

**Remady:** Thank you for this comment. We have re-analyzed the table 4. The synergetic effect of CKD and comorbidities for developing SNHL was considered when the value of HRs in patients with CKD and comorbidities (heart failure, liver cirrhosis, type 2 DM, hypertension, and COPD) got increased.

| Table 4. Factors of sensorineural hearing loss stratified by variables listed in the table by using Fine & Gray's competing risk model | | | | | | | | | | | | | |
| --- | --- | --- | --- | --- | --- | --- | --- | --- | --- | --- | --- | --- | --- |
|  |  | With CKD | | |  | Without CKD | | |  | With CKD *vs.* Without CKD *(Reference)* | | | |
| Variables | Strarified | Events | PYs | Rate |  | Events | PYs | Rate |  | Adjusted HR | 95% CI | 95% CI | *P* |
|  | Overall | 368 | 334,193.43 | 110.12 |  | 960 | 1,168,647.66 | 82.15 |  | 3.42 | 3.01 | 3.90 | <0.001^***^ |
| Gender | Male | 198 | 174,093.39 | 113.73 |  | 595 | 629,014.38 | 94.59 |  | 2.98 | 2.50 | 3.54 | <0.001^***^ |
|  | Female | 170 | 160,100.04 | 106.18 |  | 365 | 539,633.28 | 67.64 |  | 4.31 | 3.44 | 5.15 | <0.001^***^ |
| Age groups (yrs) | 18-29 | 6 | 2,450.49 | 244.85 |  | 0 | 4,350.17 | 0.00 |  | ∞ | - | - | 0.897 |
|  | 30-39 | 6 | 8,722.15 | 68.79 |  | 5 | 15,192.58 | 32.91 |  | 3.37 | 0.77 | 14.82 | 0.724 |
|  | 40-49 | 32 | 24,547.77 | 130.36 |  | 23 | 69,293.14 | 33.19 |  | 4.30 | 2.34 | 7.89 | <0.001^***^ |
|  | 50-59 | 40 | 54,197.86 | 73.80 |  | 99 | 305,306.51 | 32.43 |  | 1.61 | 1.08 | 2.41 | 0.041^*^ |
|  | ≧60 | 284 | 244,275.16 | 116.26 |  | 833 | 744,505.26 | 111.89 |  | 3.67 | 3.18 | 4.23 | <0.001^***^ |
| HTN | Without | 216 | 196,821.62 | 109.74 |  | 677 | 823,506.35 | 82.21 |  | 3.36 | 2.85 | 3.99 | <0.001^***^ |
|  | With | 152 | 137,371.81 | 110.65 |  | 283 | 345,141.31 | 82.00 |  | 3.67 | 2.95 | 4.62 | <0.001^***^ |
| T2DM | Without | 244 | 226,763.67 | 107.60 |  | 782 | 902,618.82 | 86.64 |  | 3.24 | 2.77 | 3.77 | <0.001^***^ |
|  | With | 124 | 107,429.76 | 115.42 |  | 178 | 266,028.84 | 66.91 |  | 3.98 | 3.08 | 4.85 | <0.001^***^ |
| CHF | Without | 338 | 302,066.10 | 111.90 |  | 936 | 1,085,462.92 | 86.23 |  | 3.29 | 2.87 | 3.76 | <0.001^***^ |
|  | With | 30 | 32,127.33 | 93.38 |  | 24 | 83,184.74 | 28.85 |  | 7.48 | 4.08 | 13.78 | <0.001^***^ |
| Stroke | Without | 329 | 303,790.75 | 108.30 |  | 793 | 1,031,091.16 | 76.91 |  | 3.48 | 3.03 | 4.02 | <0.001^***^ |
|  | With | 39 | 30,402.68 | 128.28 |  | 167 | 137,556.50 | 121.40 |  | 2.99 | 2.06 | 4.35 | <0.001^***^ |
| COPD | Without | 342 | 313,272.56 | 109.17 |  | 830 | 1,024,602.66 | 81.01 |  | 3.39 | 2.96 | 3.90 | <0.001^***^ |
|  | With | 26 | 20,920.87 | 124.28 |  | 130 | 144,045.00 | 90.25 |  | 3.45 | 2.23 | 5.32 | <0.001^***^ |
| Liver cirrhosis | Without | 344 | 317,482.25 | 108.35 |  | 915 | 1,108,300.52 | 82.56 |  | 3.39 | 2.97 | 3.88 | <0.001^***^ |
|  | With | 24 | 16,711.18 | 143.62 |  | 45 | 60,347.14 | 74.57 |  | 4.12 | 2.42 | 7.03 | <0.001^***^ |
| Meniere's disease | Without | 356 | 331,468.23 | 107.40 |  | 834 | 1,131,989.89 | 73.68 |  | 3.58 | 3.13 | 4.10 | <0.001^***^ |
|  | With | 12 | 2,725.20 | 440.33 |  | 126 | 36,657.77 | 343.72 |  | 1.64 | 1.01 | 3.12 | 0.047^*^ |
| Aminoglycoside | Without | 366 | 332,526.32 | 110.07 |  | 953 | 1,161,049.27 | 82.08 |  | 3.41 | 3.00 | 3.92 | <0.001^***^ |
|  | With | 2 | 1,667.11 | 119.97 |  | 7 | 7,598.39 | 92.12 |  | 3.33 | 2.83 | 3.83 | <0.001^***^ |
| Loop diuretics | Without | 367 | 331,401.74 | 110.74 |  | 957 | 1,157,557.93 | 82.67 |  | 3.42 | 3.00 | 3.93 | <0.001^***^ |
|  | With | 1 | 2,791.69 | 35.82 |  | 3 | 11,089.73 | 27.05 |  | 3.39 | 2.96 | 3.86 | <0.001^***^ |
| PYs = Person-years; Rate: per 100,000 PYs; Adjusted HR (hazard ratio): Adjusted variables listed in the table; CI = confidence interval; ^*^*P* < 0.05, ^**^*P* < 0.01, ^***^*P* < 0.001. CKD: chronic kidney disease, HTN: hypertension, T2DM: type 2 diabetes mellitus, CHF: heart failure, COPD: chronic obstructive pulmonary disease. | | | | | | | | | | | | | |

**6-6. Table 2-4**

**Combined the two columns of 95% CI.**

**Round the PYs to integer and divided 1000.**

**Round the rate, ratio (IRR), HR and 95% CI of HR to 2nd decimal place.**

**Remady:** Thank you for this comment. We has reanalyzed and revised the table 2-4.

| Table 2. Factors of sensorineural hearing loss by using Cox regression and Fine & Gray's competing risk model | | | | | | | | | |
| --- | --- | --- | --- | --- | --- | --- | --- | --- | --- |
|  | No competing risk in the model | | | |  | Competing risk in the model | | | |
|  | Adjusted HR | 95% CI | 95% CI | *P* |  | Adjusted HR | 95% CI | 95% CI | *P* |
| CKD *(Reference: Without)* | 3.19 | 2.80 | 3.64 | <0.001^***^ |  | 3.42 | 3.01 | 3.90 | <0.001^***^ |
| Gender *(Reference: Female)* | 1.30 | 1.16 | 1.45 | <0.001^***^ |  | 1.32 | 1.19 | 1.48 | <0.001^***^ |
| Age groups (yrs) *(Reference: 18-29)* |  |  |  |  |  |  |  |  |  |
| 30-39 | 0.52 | 0.19 | 1.40 | 0.192 |  | 0.53 | 0.20 | 1.43 | 0.205 |
| 40-49 | 0.95 | 0.45 | 2.21 | 0.896 |  | 0.99 | 0.42 | 2.30 | 0.969 |
| 50-59 | 0.99 | 0.44 | 2.24 | 0.968 |  | 1.05 | 0.46 | 2.37 | 0.931 |
| ≧60 | 0.12 | 0.51 | 2.53 | 0.790 |  | 1.28 | 0.57 | 2.86 | 0.568 |
| Comorbidities *(Reference: Without)* |  |  |  |  |  |  |  |  |  |
| HTN | 0.89 | 0.79 | 1.01 | 0.052 |  | 0.84 | 0.75 | 0.95 | 0.009^**^ |
| T2DM | 0.86 | 0.76 | 1.01 | 0.051 |  | 0.85 | 0.75 | 1.00 | 0.046^*^ |
| CHF | 0.52 | 0.40 | 0.69 | <0.001^***^ |  | 0.55 | 0.42 | 0.73 | <0.001^***^ |
| Stroke | 1.49 | 1.29 | 1.74 | <0.001^***^ |  | 1.52 | 1.31 | 1.77 | <0.001^***^ |
| COPD | 1.22 | 1.04 | 1.45 | 0.026 ^*^ |  | 1.21 | 1.04 | 1.53 | 0.019 ^*^ |
| Liver cirrhosis | 1.13 | 0.89 | 1.45 | 0.338 |  | 1.19 | 0.94 | 1.55 | 0.192 |
| Meniere's disease | 8.51 | 7.11 | 10.17 | <0.001^***^ |  | 7.81 | 6.53 | 9.34 | <0.001^***^ |
| CCI_R *(Reference: Without)* | 0.95 | 0.93 | 0.98 | 0.006^**^ |  | 0.97 | 0.94 | 1.00 | 0.027^*^ |
| Medications |  |  |  |  |  |  |  |  |  |
| Aminoglycoside | 0.37 | 0.12 | 1.14 | 0.267 |  | 0.37 | 0.12 | 1.18 | 0.143 |
| Loop diuretics | 0.19 | 0.05 | 0.75 | <0.001^***^ |  | 0.21 | 0.05 | 0.86 | 0.005^*^ |
| Adjusted HR (hazard ratio): Adjusted variables listed in the table; CI = confidence interval; ^*^*P* < 0.05, ^**^*P* < 0.01, ^***^*P* < 0.001. CKD: chronic kidney disease, HTN: hypertension, T2DM: type 2 diabetes mellitus, CHF: heart failure, COPD: chronic obstructive pulmonary disease. | | | | | | | | | |

| Table 3. Factors of sensorineural hearing loss stratified by with / without hemodialysis by using Cox regression and Fine & Gray's competing risk model | | | | | | | | | | | | | | |
| --- | --- | --- | --- | --- | --- | --- | --- | --- | --- | --- | --- | --- | --- | --- |
|  |  |  |  |  |  | No competing risk in the model | | | |  | Competing risk in the model | | | |
|  | Populations | Events | PYs | Rate |  | Adjusted HR | 95% CI | 95% CI | *P* |  | Adjusted HR | 95% CI | 95% CI | *P* |
| Without CKD | 556,290 | 960 | 1,168,647.66 | 82.146 |  | *Reference* |  |  |  |  | *Reference* |  |  |  |
| With CKD | 185,430 | 368 | 334,193.43 | 110.116 |  | 3.19 | 2.80 | 3.64 | <0.001^***^ |  | 3.42 | 3.01 | 3.90 | <0.001^***^ |
| without hemodialysis | 98,069 | 198 | 200,431.46 | 98.787 |  | 1.31 | 0.99 | 3.01 | 0.058 |  | 1.40 | 1.01 | 3.23 | 0.046^*^ |
| with hemodialysis | 87,361 | 170 | 133,761.97 | 127.091 |  | 5.52 | 2.82 | 10.98 | <0.001^***^ |  | 5.92 | 3.03 | 11.79 | <0.001^***^ |
| PYs = Person-years; Rate: per 100,000 PYs; Adjusted HR (hazard ratio): Adjusted variables listed in Table 1; CI = confidence interval; ^*^*P* < 0.05, ^**^*P* < 0.01, ^***^*P* < 0.001. CKD: chronic kidney disease. | | | | | | | | | | | | | | |

| Table 4. Factors of sensorineural hearing loss stratified by variables listed in the table by using Fine & Gray's competing risk model | | | | | | | | | | | | | |
| --- | --- | --- | --- | --- | --- | --- | --- | --- | --- | --- | --- | --- | --- |
|  |  | With CKD | | |  | With CKD | | |  | With CKD *vs.* Without CKD *(Reference)* | | | |
| Variables | Strarified | Events | PYs | Rate |  | Events | PYs | Rate |  | Adjusted HR | 95% CI | 95% CI | *P* |
|  | Overall | 368 | 334,193.43 | 110.12 |  | 960 | 1,168,647.66 | 82.15 |  | 3.42 | 3.01 | 3.90 | <0.001^***^ |
| Gender | Male | 198 | 174,093.39 | 113.73 |  | 595 | 629,014.38 | 94.59 |  | 2.98 | 2.50 | 3.54 | <0.001^***^ |
|  | Female | 170 | 160,100.04 | 106.18 |  | 365 | 539,633.28 | 67.64 |  | 4.31 | 3.44 | 5.15 | <0.001^***^ |
| Age groups (yrs) | 18-29 | 6 | 2,450.49 | 244.85 |  | 0 | 4,350.17 | 0.00 |  | ∞ | - | - | 0.897 |
|  | 30-39 | 6 | 8,722.15 | 68.79 |  | 5 | 15,192.58 | 32.91 |  | 3.37 | 0.77 | 14.82 | 0.724 |
|  | 40-49 | 32 | 24,547.77 | 130.36 |  | 23 | 69,293.14 | 33.19 |  | 4.30 | 2.34 | 7.89 | <0.001^***^ |
|  | 50-59 | 40 | 54,197.86 | 73.80 |  | 99 | 305,306.51 | 32.43 |  | 1.61 | 1.08 | 2.41 | 0.041^*^ |
|  | ≧60 | 284 | 244,275.16 | 116.26 |  | 833 | 744,505.26 | 111.89 |  | 3.67 | 3.18 | 4.23 | <0.001^***^ |
| HTN | Without | 216 | 196,821.62 | 109.74 |  | 677 | 823,506.35 | 82.21 |  | 3.36 | 2.85 | 3.99 | <0.001^***^ |
|  | With | 152 | 137,371.81 | 110.65 |  | 283 | 345,141.31 | 82.00 |  | 3.67 | 2.95 | 4.62 | <0.001^***^ |
| T2DM | Without | 244 | 226,763.67 | 107.60 |  | 782 | 902,618.82 | 86.64 |  | 3.24 | 2.77 | 3.77 | <0.001^***^ |
|  | With | 124 | 107,429.76 | 115.42 |  | 178 | 266,028.84 | 66.91 |  | 3.98 | 3.08 | 4.85 | <0.001^***^ |
| CHF | Without | 338 | 302,066.10 | 111.90 |  | 936 | 1,085,462.92 | 86.23 |  | 3.29 | 2.87 | 3.76 | <0.001^***^ |
|  | With | 30 | 32,127.33 | 93.38 |  | 24 | 83,184.74 | 28.85 |  | 7.48 | 4.08 | 13.78 | <0.001^***^ |
| Stroke | Without | 329 | 303,790.75 | 108.30 |  | 793 | 1,031,091.16 | 76.91 |  | 3.48 | 3.03 | 4.02 | <0.001^***^ |
|  | With | 39 | 30,402.68 | 128.28 |  | 167 | 137,556.50 | 121.40 |  | 2.99 | 2.06 | 4.35 | <0.001^***^ |
| COPD | Without | 342 | 313,272.56 | 109.17 |  | 830 | 1,024,602.66 | 81.01 |  | 3.39 | 2.96 | 3.90 | <0.001^***^ |
|  | With | 26 | 20,920.87 | 124.28 |  | 130 | 144,045.00 | 90.25 |  | 3.45 | 2.23 | 5.32 | <0.001^***^ |
| Liver cirrhosis | Without | 344 | 317,482.25 | 108.35 |  | 915 | 1,108,300.52 | 82.56 |  | 3.39 | 2.97 | 3.88 | <0.001^***^ |
|  | With | 24 | 16,711.18 | 143.62 |  | 45 | 60,347.14 | 74.57 |  | 4.12 | 2.42 | 7.03 | <0.001^***^ |
| Meniere's disease | Without | 356 | 331,468.23 | 107.40 |  | 834 | 1,131,989.89 | 73.68 |  | 3.58 | 3.13 | 4.10 | <0.001^***^ |
|  | With | 12 | 2,725.20 | 440.33 |  | 126 | 36,657.77 | 343.72 |  | 1.64 | 1.01 | 3.12 | 0.047^*^ |
| Aminoglycoside | Without | 366 | 332,526.32 | 110.07 |  | 953 | 1,161,049.27 | 82.08 |  | 3.41 | 3.00 | 3.92 | <0.001^***^ |
|  | With | 2 | 1,667.11 | 119.97 |  | 7 | 7,598.39 | 92.12 |  | 3.33 | 2.83 | 3.83 | <0.001^***^ |
| Loop diuretics | Without | 367 | 331,401.74 | 110.74 |  | 957 | 1,157,557.93 | 82.67 |  | 3.42 | 3.00 | 3.93 | <0.001^***^ |
|  | With | 1 | 2,791.69 | 35.82 |  | 3 | 11,089.73 | 27.05 |  | 3.39 | 2.96 | 3.86 | <0.001^***^ |
| PYs = Person-years; Rate: per 100,000 PYs; Adjusted HR (hazard ratio): Adjusted variables listed in the table; CI = confidence interval; ^*^*P* < 0.05, ^**^*P* < 0.01, ^***^*P* < 0.001. CKD: chronic kidney disease, HTN: hypertension, T2DM: type 2 diabetes mellitus, CHF: heart failure, COPD: chronic obstructive pulmonary disease. | | | | | | | | | | | | | |

**6-7.** **Figure 2**

**Provide number of subjects at risk below the KM curve.**

**It would be more informative if they could provide “adjusted survival curves” rather than unadjusted KM curves.**

**Remady:** Thank you for this comment. We had revised the figure 2 with number of subjects by adjusting survival curve.

Last, we are deeply honored by the time and effort you spent in reviewing this manuscript. In reviewing and revising our manuscript, we are motivated to read more and thus learn more from your criticisms.

**Response to Reviewer 2:**

**General comments:**

**In this manuscript, Wu et al used a national insurance database to retrospectively analyze the risk factors of SNHL in patients with CKD. It contains a very large cohort from the Taiwan National Health Insurance Research Database (NHIRD). They concluded that CKD patients are associated with higher risk of developing SNHL. The increased risks of SNHL were also observed in selected co-morbidities. In the manuscript, Wu et al. also included parameters such as season, locations and level of care to investigate their respective associations with SNHL. The strength of this study is its large population and duration of follow up.**

**Major concerns:**

**Point 1. This manuscript is based on the Taiwan NHIRD which is widely used for non-hypothesis driven association studies in recent years. It is well known for inaccurate disease codings and pipeline production publication. This lowers the credibility of this manuscript.**

**Remedy:** We fully understand your concern. The association between CKD and associated comorbidities had been reported in previous studies. The relation of hearing loss and CKD or other systemic diseases had also been demonstrated. We have designed the advanced study to investigate the risk of hearing loss in patients with CKD and associated comorbidities. Taiwan NHIRD was used as the database. The Bureau of National Health Insurance randomly reviews the records of 1 in 100 ambulatory care visits and 1 in 20 in-patient claims to verify the accuracy of the diagnoses. Previous studies also demonstrated the high accuracy and validity. We added “The Bureau of National Health Insurance randomly reviews the records of 1 in 100 ambulatory care visits and 1 in 20 in-patient claims to verify the accuracy of the diagnoses. The accuracy and validity of the diagnoses have been demonstrated [12,13]. Therefore, the NHIRD was used as the data source. The study used data from 2000 to 2010 from the Longitudinal NHIRD to analyze the risk of developing SNHL in patients with CKD.” (please see Material and Methods, section: Data sources, line: 109-114)

12. Cheng CL, Kao YH, Lin SJ, Lee CH, Lai ML. Validation of the National Health Insurance Research Database with ischemic stroke cases in Taiwan. Pharmacoepidemiol Drug Saf. 2011 Mar;20(3):236-42.

13.Hsieh CY, Su CC, Shao SC, Sung SF, Lin SJ, Kao Yang YH, Lai EC. Taiwan's National Health Insurance Research Database: past and future. Clin Epidemiol. 2019 May 3;11:349-358.

**Point 2. Literally, what authors presented in the manuscript were been reported in previous literatures which makes the discussion section looks like a review article. There is no novelty in this manuscript.**

**Remedy:** Thank you for your suggestion. We demonstrated the important point in discussion “Our study highlights the increased risk of developing SNHL in patients with CKD and comorbidities such as heart failure, liver cirrhosis, type 2 DM, hypertension, and COPD (table 4). Organ crosstalk is essential for maintaining physical homeostasis; however, dysfunction in one or more organs may lead to functional and structural pathological states in other organs. These medical conditions can aggravate the synergetic effect of developing SNHL by inter-organ crosstalk, which may contribute to chronic inflammation, oxidative stress, and sympathetic nerve hyperactivity [33,38,40,41,52,54]. The effective strategies in this high risk population is necessary to preserve hearing function.” (please see discussion: line: 246-254)

**Point 3. The authors did not specifically mention based on what reasons (hypothesis) did they select these co-morbidities to test the associations with SNHL, such as liver cirrhosis and COPD. Are these "selected" co-morbidities possibly just reporting bias for their p value significance?**

**Remedy:** We greatly appreciate this comment. The comorbidities were selected by in previous studies. We added the description, “Cardiovascular disease, type 2 DM and liver cirrhosis have been strongly considered as risk factors of CKD because of inflammation, ischemia, hemodynamic change, and the overactivity of renin–angiotensin–aldosterone and sympathetic nervous systems [33-40]. In recent studies, COPD may be another risk factor of CKD while it is a disease with irreversible airway obstruction and contributes to systemic inflammation that may induce vessel disease [41,42].” (please see discussion: line 233-239)

**Reference**

33.	Ronco C, Cicoira M, McCullough PA. Cardiorenal syndrome type 1: pathophysiological crosstalk leading to combined heart and kidney dysfunction in the setting of acutely decompensated heart failure. J Am Coll Cardiol. 2012;60:1031-42.

34.	Cruz DN, Bagshaw SM. Heart-kidney interaction: epidemiology of cardiorenal syndromes. Int J Nephrol. 2010;2011:351291.

35.	Persson F, Rossing P. Diagnosis of diabetic kidney disease: state of the art and future perspective. Kidney International Supplements. 2018;8:2-7.

36.	Makita Z, Radoff S, Rayfield EJ, Yang Z, Skolnik E, Delaney V, et al. Advanced glycosylation end products in patients with diabetic nephropathy. N Engl J Med. 1991;325:836-42.

37.	Nosadini R, Velussi M, Brocco E, Abaterusso C, Carraro A, Piarulli F, et al. Increased renal arterial resistance predicts the course of renal function in type 2 diabetes with microalbuminuria. Diabetes. 2006;55:234-9.

38.	Remuzzi G, Schieppati A, Ruggenenti P. Clinical practice. Nephropathy in patients with type 2 diabetes. N Engl J Med. 2002;346:1145-51.

39.	Arroyo V, Gines P, Gerbes AL, Dudley FJ, Gentilini P, Laffi G, et al. Definition and diagnostic criteria of refractory ascites and hepatorenal syndrome in cirrhosis. International Ascites Club. Hepatology. 1996;23:164-76.

40. Gines P, Schrier RW. Renal failure in cirrhosis. N Engl J Med. 2009;361:1279-90.

41. Chung-Yu Chen, Kuang-Ming Liao. Chronic Obstructive Pulmonary Disease is associated with risk of Chronic Kidney Disease: A Nationwide Case-Cohort Study. Sci Rep. 2016; 6: 25855.

42. Swarna Gaddam, Sameer K. Gunukula, James W. Lohr, Pradeep Arora. Prevalence of chronic kidney disease in patients with chronic obstructive pulmonary disease: a systematic review and meta-analysis. BMC Pulm Med. 2016;16:158.

**Point 4. It is well known that CKD patients (with or without dialysis therapy) are at higher risk of developing cardiovascular diseases. So did Wu et al. analyzed the association between stroke, CKD and SNHL. Since stroke per se would lead to SNHL independent of CKD, the authors did not adjust stroke in their analysis.**

**Remedy:** Thank your suggestion. We used adjusted HR to evaluate the risk of sensorineural hearing loss after adjusting variables listed in the table 2-4.

| Table 2. Factors of sensorineural hearing loss by using Cox regression and Fine & Gray's competing risk model | | | | | | | | | |
| --- | --- | --- | --- | --- | --- | --- | --- | --- | --- |
|  | No competing risk in the model | | | |  | Competing risk in the model | | | |
|  | Adjusted HR | 95% CI | 95% CI | *P* |  | Adjusted HR | 95% CI | 95% CI | *P* |
| CKD *(Reference: Without)* | 3.19 | 2.80 | 3.64 | <0.001^***^ |  | 3.42 | 3.01 | 3.90 | <0.001^***^ |
| Gender *(Reference: Female)* | 1.30 | 1.16 | 1.45 | <0.001^***^ |  | 1.32 | 1.19 | 1.48 | <0.001^***^ |
| Age groups (yrs) *(Reference: 18-29)* |  |  |  |  |  |  |  |  |  |
| 30-39 | 0.52 | 0.19 | 1.40 | 0.192 |  | 0.53 | 0.20 | 1.43 | 0.205 |
| 40-49 | 0.95 | 0.45 | 2.21 | 0.896 |  | 0.99 | 0.42 | 2.30 | 0.969 |
| 50-59 | 0.99 | 0.44 | 2.24 | 0.968 |  | 1.05 | 0.46 | 2.37 | 0.931 |
| ≧60 | 0.12 | 0.51 | 2.53 | 0.790 |  | 1.28 | 0.57 | 2.86 | 0.568 |
| Comorbidities *(Reference: Without)* |  |  |  |  |  |  |  |  |  |
| HTN | 0.89 | 0.79 | 1.01 | 0.052 |  | 0.84 | 0.75 | 0.95 | 0.009^**^ |
| T2DM | 0.86 | 0.76 | 1.01 | 0.051 |  | 0.85 | 0.75 | 1.00 | 0.046^*^ |
| CHF | 0.52 | 0.40 | 0.69 | <0.001^***^ |  | 0.55 | 0.42 | 0.73 | <0.001^***^ |
| Stroke | 1.49 | 1.29 | 1.74 | <0.001^***^ |  | 1.52 | 1.31 | 1.77 | <0.001^***^ |
| COPD | 1.22 | 1.04 | 1.45 | 0.026 ^*^ |  | 1.21 | 1.04 | 1.53 | 0.019 ^*^ |
| Liver cirrhosis | 1.13 | 0.89 | 1.45 | 0.338 |  | 1.19 | 0.94 | 1.55 | 0.192 |
| Meniere's disease | 8.51 | 7.11 | 10.17 | <0.001^***^ |  | 7.81 | 6.53 | 9.34 | <0.001^***^ |
| CCI_R *(Reference: Without)* | 0.95 | 0.93 | 0.98 | 0.006^**^ |  | 0.97 | 0.94 | 1.00 | 0.027^*^ |
| Medications |  |  |  |  |  |  |  |  |  |
| Aminoglycoside | 0.37 | 0.12 | 1.14 | 0.267 |  | 0.37 | 0.12 | 1.18 | 0.143 |
| Loop diuretics | 0.19 | 0.05 | 0.75 | <0.001^***^ |  | 0.21 | 0.05 | 0.86 | 0.005^*^ |
| Adjusted HR (hazard ratio): Adjusted variables listed in the table; CI = confidence interval; ^*^*P* < 0.05, ^**^*P* < 0.01, ^***^*P* < 0.001. CKD: chronic kidney disease, HTN: hypertension, T2DM: type 2 diabetes mellitus, CHF: heart failure, COPD: chronic obstructive pulmonary disease. | | | | | | | | | |

| Table 3. Factors of sensorineural hearing loss stratified by with / without hemodialysis by using Cox regression and Fine & Gray's competing risk model | | | | | | | | | | | | | | |
| --- | --- | --- | --- | --- | --- | --- | --- | --- | --- | --- | --- | --- | --- | --- |
|  |  |  |  |  |  | No competing risk in the model | | | |  | Competing risk in the model | | | |
|  | Populations | Events | PYs | Rate |  | Adjusted HR | 95% CI | 95% CI | *P* |  | Adjusted HR | 95% CI | 95% CI | *P* |
| Without CKD | 556,290 | 960 | 1,168,647.66 | 82.146 |  | *Reference* |  |  |  |  | *Reference* |  |  |  |
| With CKD | 185,430 | 368 | 334,193.43 | 110.116 |  | 3.19 | 2.80 | 3.64 | <0.001^***^ |  | 3.42 | 3.01 | 3.90 | <0.001^***^ |
| without hemodialysis | 98,069 | 198 | 200,431.46 | 98.787 |  | 1.31 | 0.99 | 3.01 | 0.058 |  | 1.40 | 1.01 | 3.23 | 0.046^*^ |
| with hemodialysis | 87,361 | 170 | 133,761.97 | 127.091 |  | 5.52 | 2.82 | 10.98 | <0.001^***^ |  | 5.92 | 3.03 | 11.79 | <0.001^***^ |
| PYs = Person-years; Rate: per 100,000 PYs; Adjusted HR (hazard ratio): Adjusted variables listed in Table 1; CI = confidence interval; ^*^*P* < 0.05, ^**^*P* < 0.01, ^***^*P* < 0.001. CKD: chronic kidney disease. | | | | | | | | | | | | | | |

| Table 4. Factors of sensorineural hearing loss stratified by variables listed in the table by using Fine & Gray's competing risk model | | | | | | | | | | | | | |
| --- | --- | --- | --- | --- | --- | --- | --- | --- | --- | --- | --- | --- | --- |
|  |  | With CKD | | |  | Without CKD | | |  | With CKD *vs.* Without CKD *(Reference)* | | | |
| Variables | Strarified | Events | PYs | Rate |  | Events | PYs | Rate |  | Adjusted HR | 95% CI | 95% CI | *P* |
|  | Overall | 368 | 334,193.43 | 110.12 |  | 960 | 1,168,647.66 | 82.15 |  | 3.42 | 3.01 | 3.90 | <0.001^***^ |
| Gender | Male | 198 | 174,093.39 | 113.73 |  | 595 | 629,014.38 | 94.59 |  | 2.98 | 2.50 | 3.54 | <0.001^***^ |
|  | Female | 170 | 160,100.04 | 106.18 |  | 365 | 539,633.28 | 67.64 |  | 4.31 | 3.44 | 5.15 | <0.001^***^ |
| Age groups (yrs) | 18-29 | 6 | 2,450.49 | 244.85 |  | 0 | 4,350.17 | 0.00 |  | ∞ | - | - | 0.897 |
|  | 30-39 | 6 | 8,722.15 | 68.79 |  | 5 | 15,192.58 | 32.91 |  | 3.37 | 0.77 | 14.82 | 0.724 |
|  | 40-49 | 32 | 24,547.77 | 130.36 |  | 23 | 69,293.14 | 33.19 |  | 4.30 | 2.34 | 7.89 | <0.001^***^ |
|  | 50-59 | 40 | 54,197.86 | 73.80 |  | 99 | 305,306.51 | 32.43 |  | 1.61 | 1.08 | 2.41 | 0.041^*^ |
|  | ≧60 | 284 | 244,275.16 | 116.26 |  | 833 | 744,505.26 | 111.89 |  | 3.67 | 3.18 | 4.23 | <0.001^***^ |
| HTN | Without | 216 | 196,821.62 | 109.74 |  | 677 | 823,506.35 | 82.21 |  | 3.36 | 2.85 | 3.99 | <0.001^***^ |
|  | With | 152 | 137,371.81 | 110.65 |  | 283 | 345,141.31 | 82.00 |  | 3.67 | 2.95 | 4.62 | <0.001^***^ |
| T2DM | Without | 244 | 226,763.67 | 107.60 |  | 782 | 902,618.82 | 86.64 |  | 3.24 | 2.77 | 3.77 | <0.001^***^ |
|  | With | 124 | 107,429.76 | 115.42 |  | 178 | 266,028.84 | 66.91 |  | 3.98 | 3.08 | 4.85 | <0.001^***^ |
| CHF | Without | 338 | 302,066.10 | 111.90 |  | 936 | 1,085,462.92 | 86.23 |  | 3.29 | 2.87 | 3.76 | <0.001^***^ |
|  | With | 30 | 32,127.33 | 93.38 |  | 24 | 83,184.74 | 28.85 |  | 7.48 | 4.08 | 13.78 | <0.001^***^ |
| Stroke | Without | 329 | 303,790.75 | 108.30 |  | 793 | 1,031,091.16 | 76.91 |  | 3.48 | 3.03 | 4.02 | <0.001^***^ |
|  | With | 39 | 30,402.68 | 128.28 |  | 167 | 137,556.50 | 121.40 |  | 2.99 | 2.06 | 4.35 | <0.001^***^ |
| COPD | Without | 342 | 313,272.56 | 109.17 |  | 830 | 1,024,602.66 | 81.01 |  | 3.39 | 2.96 | 3.90 | <0.001^***^ |
|  | With | 26 | 20,920.87 | 124.28 |  | 130 | 144,045.00 | 90.25 |  | 3.45 | 2.23 | 5.32 | <0.001^***^ |
| Liver cirrhosis | Without | 344 | 317,482.25 | 108.35 |  | 915 | 1,108,300.52 | 82.56 |  | 3.39 | 2.97 | 3.88 | <0.001^***^ |
|  | With | 24 | 16,711.18 | 143.62 |  | 45 | 60,347.14 | 74.57 |  | 4.12 | 2.42 | 7.03 | <0.001^***^ |
| Meniere's disease | Without | 356 | 331,468.23 | 107.40 |  | 834 | 1,131,989.89 | 73.68 |  | 3.58 | 3.13 | 4.10 | <0.001^***^ |
|  | With | 12 | 2,725.20 | 440.33 |  | 126 | 36,657.77 | 343.72 |  | 1.64 | 1.01 | 3.12 | 0.047^*^ |
| Aminoglycoside | Without | 366 | 332,526.32 | 110.07 |  | 953 | 1,161,049.27 | 82.08 |  | 3.41 | 3.00 | 3.92 | <0.001^***^ |
|  | With | 2 | 1,667.11 | 119.97 |  | 7 | 7,598.39 | 92.12 |  | 3.33 | 2.83 | 3.83 | <0.001^***^ |
| Loop diuretics | Without | 367 | 331,401.74 | 110.74 |  | 957 | 1,157,557.93 | 82.67 |  | 3.42 | 3.00 | 3.93 | <0.001^***^ |
|  | With | 1 | 2,791.69 | 35.82 |  | 3 | 11,089.73 | 27.05 |  | 3.39 | 2.96 | 3.86 | <0.001^***^ |
| PYs = Person-years; Rate: per 100,000 PYs; Adjusted HR (hazard ratio): Adjusted variables listed in the table; CI = confidence interval; ^*^*P* < 0.05, ^**^*P* < 0.01, ^***^*P* < 0.001. CKD: chronic kidney disease, HTN: hypertension, T2DM: type 2 diabetes mellitus, CHF: heart failure, COPD: chronic obstructive pulmonary disease. | | | | | | | | | | | | | |

**Point 5. It seems that seasons, location, urbanization and level of care are also associated with SNHL. These factors were adjusted but not explained the reason of significance between them and SNHL? Are these statistical significance or any biological significance involved?**

**Remedy:** Thank you for the reminder. We deleted these factors (s**easons, location, urbanization and level of care**) because of no biological significance between them and SNHL.

**Point 6. The discussion section looks like a review article but not much of new information or specifically based on authors' findings.**

**Remedy:** Thank your command. Previous reports have descripted that CKD patients often have associated comorbidities. For sensorineural hearing loss, the risk of some diseases has been evaluated. Our study highlights the increased risk of developing sensorineural hearing loss in patients with CKD and comorbidities such as heart failure, liver cirrhosis, type 2 DM, hypertension, and COPD. Based on our results, the development of effective strategies in this high risk population is necessary to preserve hearing function.

(Please see Discussion, 2nd paragraph)

**Minor concerns:**

**Point 1. The first sentence in the discussion, it is not correct to use "prospectively" since this was a retrospective study.**

**Remedy:** Thank your suggestion. We deleted prospectively in first sentence and changed “In this study, a large cohort of patients with newly diagnosed CKD was evaluated for the risk of developing SNHL.”(discussion: line 204-205)

Last, I am deeply honored by the time and effort you spent in reviewing this manuscript. In reviewing and revising our manuscript, I was motivated to read more and thus learn more from your criticisms.
